# Boolean Models of Biosurfactants Production in *Pseudomonas fluorescens*

**DOI:** 10.1371/journal.pone.0024651

**Published:** 2012-01-31

**Authors:** Adrien Richard, Gaelle Rossignol, Jean-Paul Comet, Gilles Bernot, Jannine Guespin-Michel, Annabelle Merieau

**Affiliations:** 1 Laboratoire d'Informatique, Signaux et Systèmes Complexes de Sophia-Antipolis (I3S), Université de Nice-Sophia Antipolis and Centre National de la Recherche Scientifique (CNRS), Sophia-Antipolis, France; 2 Laboratoire de Microbiologie du Froid - Signaux et Micro-Environnement (LMDF-SME), Université de Rouen, Evreux, France; Semmelweis University, Hungary

## Abstract

Cyclolipopeptides (CLPs) are biosurfactants produced by numerous *Pseudomonas fluorescens strains*. CLP production is known to be regulated at least by the GacA/GacS two-component pathway, but the full regulatory network is yet largely unknown. In the clinical strain MFN1032, CLP production is abolished by a mutation in the phospholipase C gene (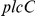) and not restored by 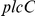 complementation. Their production is also subject to phenotypic variation. We used a modelling approach with Boolean networks, which takes into account all these observations concerning CLP production without any assumption on the topology of the considered network. Intensive computation yielded numerous models that satisfy these properties. All models minimizing the number of components point to a bistability in CLP production, which requires the presence of a yet unknown key self-inducible regulator. Furthermore, all suggest that a set of yet unexplained phenotypic variants might also be due to this epigenetic switch. The simplest of these Boolean networks was used to propose a biological regulatory network for CLP production. This modelling approach has allowed a possible regulation to be unravelled and an unusual behaviour of CLP production in *P. fluorescens* to be explained.

## Introduction

*Pseudomonas fluorescens* are ubiquitous bacteria endowed with a capacity to adapt to many environments, namely by producing a wide range of extracellular molecules such as enzymes, toxins, antibiotics and biosurfactants. In these species, the main characterized biosurfactants are cyclolipopeptides (CLPs). They are involved in swarming motility and, depending on the strain and/or on the nature the considered CLPs, are sometimes required for biofilm formation [1].

Up to now studies have mainly focused on the effects of environmental conditions on CLP production (temperature, substrate, pH…), but data concerning transduction of the signals are quite limited [2]. Some regulators are involved in CLP production control in all *Pseudomonas* species, whereas others seem to be species dependent or even strain dependent. The global regulatory system GacS/GacA controls CLP production at a post-transcriptional level by the Rsm pathway in all *Pseudomonas* spp. [3]–[5]. On the other hand, in *P. fluorescens*, the requirement of the N-acylhomoserine lactone mediated quorum sensing for CLP production has only been demonstrated in a few strains [6]. In *P. fluorescens* SS101 and SBW25 (strains that do not produce N-acylhomoserine lactone), the LuxR-type transcriptional regulator positively regulates the production of CLPs. These LuxR-type regulators do not harbour the autoinducer-binding domain found in most quorum sensing associated LuxR regulators. Lux R-type genes are under the control of both GacS/GacA, and of the serine protease ClpP in a GacS/GacA-independent way [5].

In a previous study [7], we showed that the *P. fluorescens* clinical strain MFN1032 displays hemolytic activity due both to phospholipase C (PlcC) and CLPs. A knock out mutation in gene 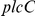 leads to the expected loss in hemolytic activity, but also to the unexpected loss of CLPs. Furthermore, and even more unexpectedly, complementation of the 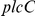 mutant [7] restored phospholipase C activity, but not the production of CLPs. We suggested that a not yet identified regulation takes place between PlcC and CLPs. In addition, CLP production in MFN1032 is subjected to phenotypic variation. After several generations, in specific conditions at 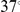
C, 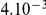 variants per generation are no longer able to produce CLPs. Fifty percent of these variants are mutants in the gacA/gacS genes but 50% are not [8].

Heterogeneity within a bacterial population is a well-known phenomenon, which supposedly allow bacteria to invade a wide range of ecosystems and increases the overall fitness of the species. The heterogeneity could result from genetic rearrangement (high rate mutations or inversions of a DNA segment), or DNA methylation, but also to epigenetic switches [9]. Thomas argued that a positive feedback circuit (that may be the self-stimulation of a pivotal transcription factor) is always necessary for such switches [10]. This property has been proved mathematically in continuous [11], Boolean [12] and discrete [13] dynamic frameworks. This property has been documented in numerous bacterial regulatory systems, and demonstrated in some of them using mathematical modelling of the network's dynamics. In a previous study [14], we demonstrated, using Thomas generalised logic modelling followed by experiments suggested by the model, that the Type 3 secretion system (T3SS) in *Pseudomonas aeruginosa* is subjected to an epigenetic switch between a non-inducible and an inducible state. The positive feedback is due to the self-regulating gene *exsA*, a general positive regulator of the regulon. More recently, MacLeane and Studholme [15] used a Boolean model to predict the dynamic behaviour of the regulation of the T3SS in *Pseudomoans syringae*, displaying a bistability under the control of the GacA/GacS node.

Here we used a Boolean approach to construct all the minimal regulatory networks of CLP production, consistent with the observations previously reported. We show that the simplest explanation for these behaviours is an epigenetic switch of the production of CLPs, the pivotal self regulating gene being unknown.

## Methods

### 2.1 Boolean network

A *Boolean network* with 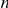 components is here defined as function 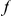 associating with each vector 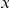 of 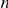 Boolean components a vector 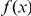 of 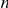 Boolean components:
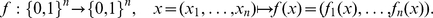
In the context of genetic regulatory systems, each component 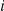 is seen as a gene that encodes a protein 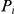 (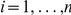). The vector 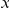 describes the state of the system in terms of the presence or absence of the proteins: protein 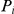 is present if 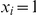, and absent if 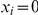. Function 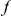 describes the activity of the genes according to the state 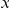 of the system: gene 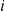 is “on” if 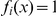, and “off” if 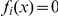. The evolution of the system is driven by the expression of the genes: if 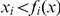 then 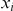 is called to increase (protein absent, gene “on”); if 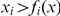 then 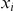 is called to decrease (protein present, gene “off”); and if 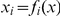, then 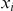 is not called to change.

### 2.2 Asynchronous state graph

As argued by Thomas, the appearance or disappearance of a protein occurs after a vacillating delay [16]. As a consequence, the simultaneously updating of several variables implies unlikely equalities between delays. It is thus reasonable to assume that at most one variable is updated at each step (this is the so called asynchronous hypothesis). Without additional information on the delays, this leads to indecision in the variable to update, and all the possibilities are considered (if 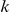 variables are called to change, then 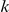 next states are possible). Formally, as in Thomas' logical method [16], the dynamic of network 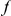 is described by a directed graph, called the *asynchronous state graph* of 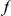: the vertex set is the set of states 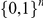; for each state 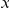 and each component 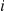 such that 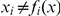, there is an arc (or transition), from state 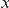 to state 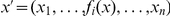.

### 2.3 Attractors

If 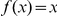, then 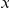 has no outgoing arc in the asynchronous state graph, and it corresponds to a *stable state* of the network. More generally, an *attractor* is a smallest non-empty subset of states that we cannot leave by following the paths of the state graph (stable states are attractors of size one). If we assume that, at each time 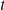, each transition starting from the current state 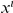 can be taken with a probability strictly greater than 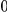, then, the probability that 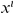 belongs to an attractor tends toward 1 when 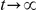. Then, one can consider that, at equilibrium, the system is always inside an attractor.

### 2.4 Interaction graph

The *interaction graph* of 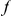, which describes the structure of the network, is the signed directed graph defined by: the vertices are the components 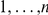, and there is a positive (resp. negative) arc 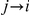 if there is a state 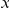 such that the difference
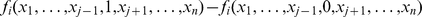
is positive (resp. negative). Hence, there is an interaction from 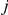 to 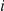 whenever function 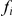 depends on variable 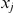, that is, whenever the activity of gene 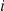 depends on the level of protein 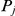. Actually, the definition says that a positive (resp. negative) interaction from 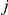 to 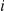 exists if, in at least one state 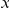, the appearance of 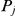 turns on (resp. turns off) gene 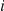. It is possible that one may have both a positive and a negative interaction from 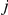 to 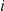 (in such a case, the sign of the interaction depends on the state of the system).

### 2.5 Muted network

We denote by 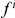 the Boolean networks with 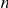 components defined by
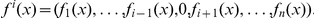
Therefore, 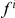 and 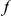 are identical except that, in 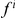, gene 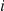 is always “off”. One can see 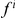 as the Boolean network resulting from a knock-out mutation of 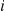 in network 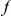. Naturally, the interaction graph of the muted network 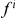 is obtained from the one of 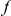 by deleting all the incoming interactions of 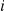.

### 2.6 Computation of consistent networks

In the next section, we are interested in the set of all the minimal Boolean networks whose asynchronous state graph satisfies some properties extracted from biological observations. To compute these networks, we used SMBioNet software [17], [18], which is dedicated to the generation and verification of discrete dynamical systems. For instance, this tool can take as input an interaction graph 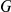 and a temporal property 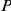 written in the Computational Tree Logic (CTL), and return the set of all the Boolean networks 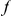 such that the interaction graph of 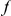 is a subgraph of 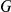, and such that the asynchronous state graph of 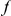 satisfies property 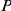. In particular, if 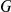 is the complete interaction graph on 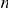 vertices, then the output consists in all the Boolean networks with 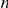 components whose asynchronous state graph satisfies property 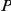. However, since the number of Boolean networks with 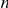 components is 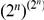, i.e. over exponential with 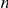, this works only for very small 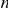 values, typically 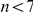.

## Results and Discussion

### 3.1 Biological connections between CLPs and PlcC

Table 1 summarizes the results obtained from our previous studies on the role of a phospholipase C in the hemolytic activity of *P. fluorescens* clinical strain MFN1032. This strain displayed hemolytic activity (HA) and PlcC and CLPs (which we previously showed were necessary for hemolytic activity). A mutation in 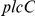 abolished not only the hemolytic activity, but also CLP production, whereas complementation of gene 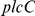 (by introducing a plasmid carrying the 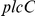 gene under the control of a weak promoter in strain MFN1037) restored PlcC production but not CLPs. The same phenotype was observed after reversion of the PlcC deficient mutant.

**Table 1:** Summary of experimental results concerning the production of hemolytic activity, phospholipase C and cyclolipopeptides in different genetic backgrounds.

Strains of Pseudomonas fluorescens	Hemolytic activity (HA)	Phospholipase (PlcC)	Cyclolipopeptides (CLPs)
MFN1032 (WT)	+	+	+
MFN1037 ( 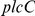 mutant)	−	−	−
MFN1038 ( 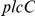 mutant complemented with 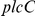 )	−	+	−
MFN1032 Group 1 variants	−	−	−
MFN1032 (Group 1 variants complemented by 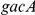 or 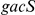 gene)	+	+	+
MFN1032 Group 2 variants	−	+	−

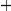
: Presence; 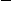: Absence (early stationary growth phase in LB medium at 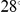
C).

How can we explain this surprising, but reproducible, process? The aim of this section is to propose a partial answer by exhibiting Boolean models that “explain” the mechanism underlying this process by the presence of genetic regulatory systems displaying two attractors, which describe an epigenetic switch. For that, we proceeded in two steps. First, we interpreted the previous observations in terms of Boolean networks, and then, we exhibited Boolean networks that were consistent with this interpretation.

#### 3.1.1 Interpretation in terms of Boolean networks of connections between CLPs and PlcC

For the interpretation, we used the following hypotheses. (1) The production of CLPs in strain MFN1032 is controlled by an asynchronous Boolean network 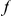, which contains at least two components: a component 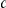 for 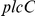, and a component 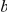 for the production of CLPs. (2) The production of CLPs in a 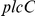 mutant (strain MFN1037) is controlled by Boolean network 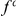. (3) The production of CLPs in the complemented 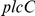 mutant (strain MFN1038) is controlled by the initial Boolean network 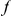. In other words, we assume that the complementation of 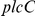 mutation restores the WT network. (4) The experimental results have been obtained at equilibrium, where the current state of network 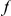 is inside an attractor.

Table 1 shows that MFN1032 displays hemolytic activity, and both PlcC and CLPs. The interpretation is that, in MFN1032, the current state of network 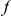 belongs to an attractor 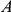 such that 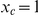 and 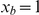 for every state 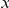 in 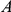. The fact that a mutation of 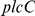 abolishes CLP production is then interpreted as follows: originally, the state of the network is in attractor 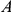, and a 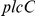 mutation leads the system to an attractor in which CLPs are absent. Hence, the asynchronous state graph of muted network 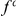 contains a path from 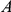 to an attractor 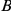 such that 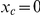 for every state 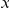 in 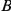. Finally, the fact that complementation of 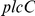 mutation restores PlcC production, but not for CLPs', is interpreted as follows: once the 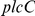 mutation has led the network to attractor 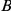, a complementation of the mutation leads the network to an attractor in which PlcC is present and CLPs are absent. Therefore, the asynchronous state graph of 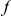 contains a path from 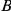 to an attractor 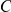 such that 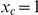 and 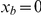 for every state 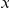 in 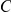.

To be consistent with this interpretation of the results, 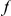 has to satisfy the following property 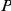:

*The asynchronous state graph of* 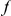 *contains an attractor* 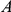 *such that: (1)* 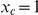 *and* 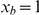 *for every state* 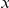 *in* 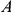 *(MFN1032); (2) the asynchronous state graph of* 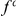 *contains a path from* 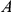 *to an attractor* 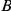 *such that* 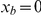 *for every state* 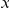 *in* 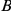 *(MFN1037); and (3) the asynchronous state graph of* 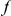 *contains a path from* 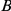 *to an attractor* 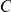 *such that* 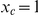 *and* 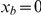 *for every state* 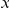 *in* 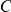 *(MFN1038).*

#### 3.1.2 Boolean networks for connections between CLPs and PlcC

Now, we must determine whether Boolean networks exist which satisfy property 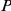. First we observe that property 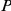 implies that the asynchronous state graph of 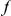 has at least two distinct attractors 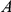 and 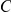 such that 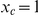 for all states 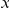 in 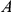 or 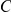. It has been proved that such a property requires the presence, in the interaction graph of 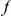, of at least one positive circuit, which does not contain vertex c (a positive circuit is a directed cycle with an even number of negative arcs and without repeated vertexes). This theorem is known as the Boolean version of the first Thomas' conjecture [12]. Since it is accepted that CLPs do not have regulatory activity, the interaction graph of a consistent network 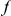 cannot have interactions starting from 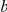. So the required positive circuit of the interaction graph of 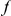 contains neither 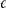 nor 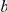. This means that at least a third component, say 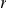, is needed.

Using intensive computation, we have generated the 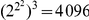 Boolean networks with three variables (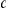, 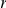, 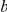) such that 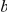 has no outgoing interaction, and demonstrated (by model checking) that exactly 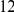 of them satisfy property 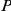 (this generation/selection procedure takes less than one minute on a common laptop, with 2.5 GHz CPU and 4 GB RAM). These 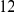 consistent Boolean networks are given in Figure S1. In all cases, the interaction graph contains a positive arc from 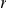 to itself, and this positive loop on 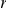 corresponds to the required positive circuit. Furthermore, there is always a positive path from 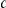 to 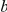, and this is consistent with the fact that the experimental results show that a mutation in 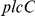 abolishes CLP production.

**Figure 1:**
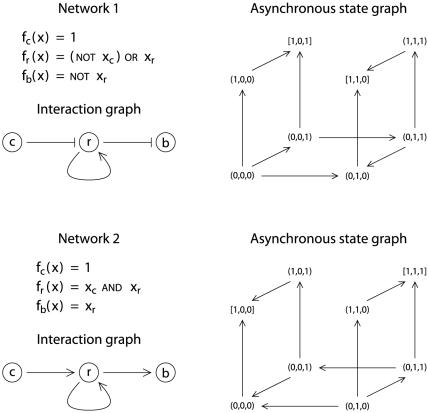
The two minimal consistent Boolean networks with 3 components. The graphical conventions used to described the interaction graph of each network are as follows: normal arrows correspond to positive interactions, and T-end arrows correspond to negative interactions. Asynchronous state graphs are represented in a 3D-grid, with 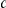 in the vertical axis (first component), 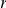 in the horizontal axis (second component), and 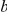 in the third axis (third component).

**Figure 2:**
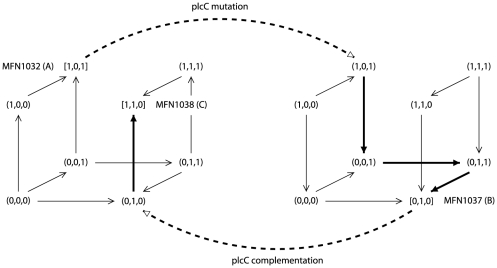
Illustration of property 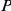
 **on network 1.** Asynchronous state graphs are represented in a grid, as in Figure 1. The left graph described the dynamics of network 1. The right graph described the dynamics of network 1 under 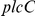 mutation. Stable states 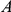, 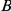 and 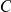 correspond to strains MFN1032, MFN1037 and MFN 1038, respectively. Dotted arrows represent the effect of mutation or complementation in 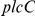. Bold lines represent trajectories followed by the systems.

There are two minimal consistent Boolean networks with respect to the number of interactions. These two networks contain three interactions, and are shown in Figure 1. According to network 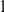 of Figure 1, 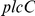 is always “on”, gene 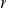 is “on” if its own product is present or if PlcC is absent, and CLPs are produced when the product of 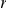 is absent. Additionally, attractor 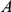 corresponds to stable state 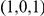, and attractor 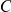 corresponds to stable state 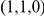. Initially, the network is in stable state 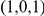, which is consistent with the phenotype of MFN1032 (PlcC and CLPs are present). Then, as illustrated in Figure 2, a mutation in 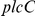 induces, firstly a disappearance of PlcC, and then an increase of 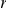 (due to the negative interaction from 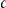 to 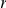) that causes a disappearance of CLPs (triggered by the negative interaction from 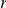 to 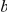). These three changes lead the network in attractor 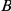, which here corresponds to stable state 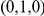, and which is consistent with the phenotype of MFN1037 (PlcC and CLPs are absent). Finally, complementation of 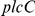 restores the production of PlcC, but the positive loop on 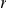 allows 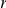 to remain present, and as a consequence, CLPs remain absent. The network then remains in stable state 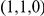, which is consistent with the phenotype of MFN1038.

Network 2 could be discussed similarly. 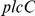 is always “on”, gene 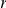 is “on” if its own product is present and if PlcC is present, and CLPs are produced when the product of 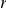 is present. In addition, attractor 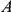 corresponds to stable state 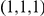, and attractor 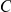 corresponds to stable state 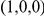. Initially, the network is in stable state 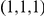, which is consistent with the phenotype of MFN1032 (PlcC and CLPs are present). A mutation in 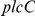 induces, firstly a disappearance of PlcC, and then a disappearance of 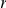 that induces a disappearance of CLPs. These three changes lead the network in attractor 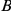, which here corresponds to stable state 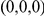, and which is consistent with the phenotype of MFN1037 (PlcC and CLPs are absent). Finally, complementation of 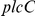 restores the production of PlcC, but 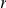 remains absent (PlcC alone is not sufficient to restore 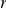 expression), and as a consequence, CLPs remain absent. The network then remains in stable state 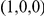, which is consistent with the phenotype of MFN1038.

Since PlcC is absent in the early growth phase and is not sufficient to restore 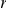 expression, this model seems less likely from a biological point of view, so we shall only discuss Network 1 in the following sections.

### 3.2 Connections between CLPs, PlcC and the GacS/GacA system

In a previous study, we have shown that strain MFN1032 displays phenotypic variations when incubated in specific conditions. The results are also summarized in Table 1.

Frequently, 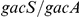 mutants occur (as in other *Pseudomonas* spp.) and generate group 1 variants, where the WT phenotype is restored by complementation by one of the two genes encoding the Gac system. A second class of variants, group 2, is composed of variants, which are not restored to WT by complementation by the 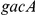 or 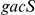 genes. Group 2 variants have a phenotype similar to the 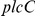 complemented mutant (strain MFN1038 or to the reverted 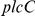 mutant) with respect to PlcC and CLP production. They neither produced CLPs nor hemolytic activity. The mechanism involved in this variation has not been elucidated [8].

#### 3.2.1 Interpretation in terms of Boolean networks of connections between CLPs, PlcC and the GacS/GacA system

For the interpretation of group 1 variants, we consider a network 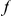 with at least three components: 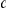, 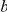, and an additional component 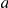 for the 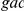 gene. The modelling hypotheses we used are similar (in particular, the production of CLPs in a 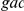 mutant is controlled by 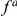, and under a complementation of 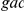 mutation, it is controlled by the initial Boolean network 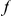).

As previously demonstrated, the WT phenotype is interpreted by the presence, in the asynchronous state graph of 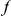, of an attractor 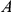 such that 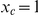 and 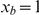 for all states 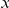 in 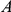. Resulting in the interpretation of the group 1 phenotype: originally, the state of the system is in 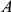, and a 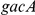 mutation leads the system to an attractor in which PlcC and CLPs are absent. The asynchronous state graph of 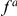 contains a path from 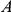 to an attractor 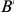 such that 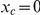 and 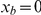 for all states 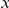 in 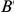. Finally, the fact that a complementation of a mutation in 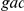 restores the wild type phenotype in group 1 variants is interpreted as follows: once the 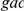 mutation has led the system to attractor 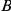, a complementation of the mutation leads the system to an attractor in which PlcC and CLPs are present. The asynchronous state graph of 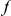 then contains a path from 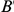 to an attractor 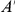 such that 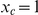 and 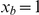 for every 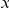 in 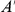.

To be consistent with both this interpretation of the 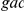 gene influence and the previous property 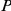, the hypothetical network 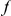 has to satisfy the following property 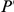:

*The asynchronous state graph of* 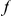 *contains an attractor* 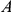 *verifying points (1–3) of property* 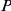 *, and two additional points: (4) the asynchronous state graph of* 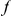 *contains a path from* 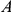 *to an attractors* 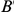 *such that* 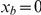 *and* 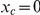 *for all states* 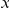 *in* 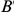 *(Group 1); (5) the asynchronous state graph of* 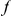 *contains a path from* 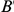 *to an attractor* 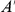 *such that* 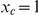 *and* 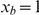 *for all states* 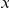 *in* 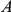 *(complementation of* 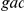 *gene in Group 1).*

#### 3.2.2 Boolean networks for connections between CLPs, PlcC and the GacS/GacA system

We now exhibit Boolean networks satisfying property 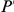. We assume that 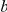 has no outgoing interaction (as previously stated), and that 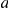 is an input of the system, i.e., has no incoming interaction. Since property 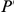 implies property 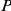, in order to satisfy 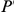, the interaction graph of 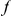 has to contain a positive circuit that does not contain vertex 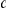. Since neither 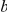 nor 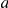 belong to a circuit (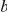 has no outgoing interaction, and 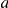 no incoming interaction), the additional component 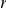 is again needed.

**Figure 3:**
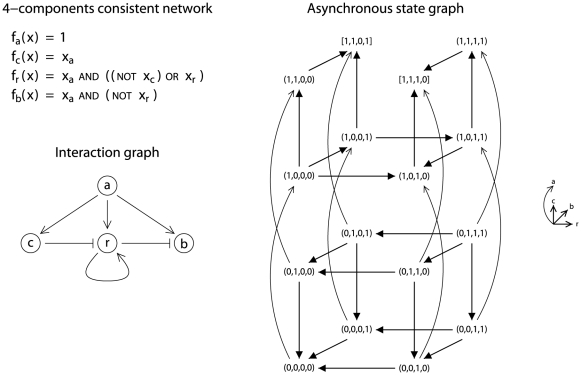
A minimal consistent Boolean network with 4 components. The asynchronous state graph is represented in a 4D-grid, with 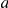 in the curved axis (first component), 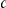 in the vertical axis (second component), 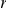 in the horizontal axis (third component), and 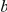 in the last axis (fourth component).

**Figure 4:**
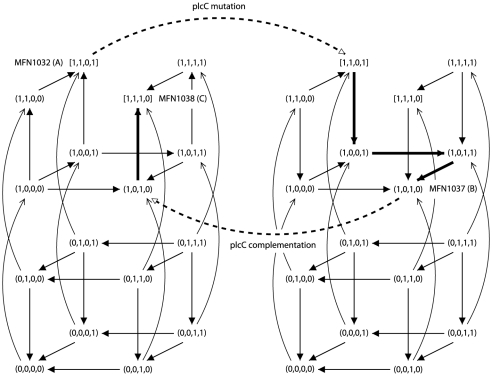
Illustration of property 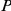
 **on the consistent network of** **Figure 3** **.** Asynchronous state graphs are represented in a 4D-grid, as in Figure 3. The left graph described the dynamics of network of Figure 3. The right graph described the dynamics of this network under 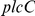 mutation. Stable states 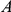, 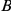 and 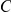 corresponds to strains MFN1032, MFN1037 and MFN1038, respectively. Dotted arrows represent the effect of mutation or complementation in 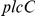. Bold lines represent trajectories followed by the systems.

**Figure 5:**
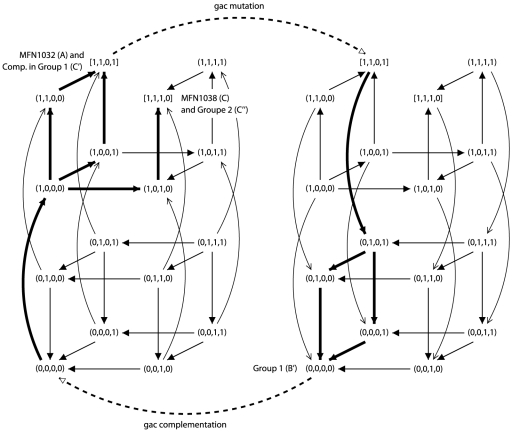
Illustration of property 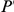
 **on the consistent network of** **Figure 3** **.** Asynchronous state graphs are represented in a 4D-grid, as in Figure 3. The left graph described the dynamics of network of Figure 3. The right graph described the dynamics of this network under 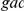 mutation. Stable states 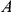 and 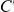 correspond to strains MFN1032 and group 1 variants complemented by 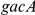 or 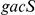 gene, stable state 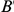 corresponds to group 1 variants, and stable states 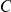 corresponds to strains MFN1038 and group 2 variants. Dotted arrows represent the effect of mutation or complementation in 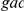. Bold lines represent trajectories followed by the systems.

Using intensive computation, as previously done, we have generated, among the among the 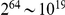 Boolean networks with four components (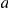, 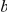, 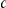, 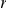), the 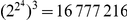 Boolean networks such that 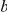 has no outgoing interaction, and such that 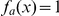 (this is clearly a necessary condition for satisfying 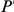). Using model checking, we show that exactly 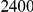 of them satisfy 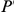 (this generation/selection procedure takes about 82 hours on an usual laptop, with 2.5 GHz CPU and 4 GB RAM). The asynchronous state graph of each consistent network contains exactly two attractors, which are stable states. Furthermore, the interaction graph of each consistent network contains at least 6 interactions, and 12 consistent networks have an interaction graph with exactly 6 interactions. One of them is given in Figure 3. We only discuss this minimal consistent network since it can be seen as an extension of Network 1 given in Figure 1 (and, as argued in the previous section, this network is the most likely minimal 3-component network). Indeed, we can see that, in the hyper-plane “
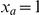
” (i.e. in the “top 3-dimensional cube”) the dynamics of the network is the one of Network 1 given in Figure 1. As a consequence, the 4-component network is consistent with property 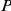, as illustrated in Figure 4. Additionally, a 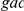 mutation leads the network to stable state 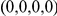 where no product is present, which is consistent with group 1 phenotype, see Figure 5. Then, complementation (or reversion) of 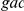 leads the network to the state 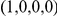 from which the original stable state 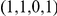, which is consistent with both the WT and the complemented group 1 phenotypes, can be reached. But from state 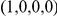, a path leading to stable state 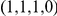 also exists, which is consistent with both the complemented plcC mutant and group 2 phenotypes. According to this model, group 2 variants could be due to a 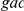 mutation followed by a reversion. This is not unlikely since 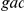 mutations and reversions are frequent in Pseudomonas spp [19].

Surprisingly, each of the 2400 consistent Boolean networks is in agreement with this hypothesis. Indeed, for each consistent Boolean network, once a 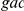 mutation has led the network in attractor 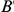, a complementation induces two possible futures: either (1) the system reaches attractor 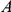, which is consistent with both the WT and the complemented group 1 phenotypes, or (2) the system reaches attractor 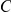, which is consistent with both the complemented 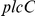 mutant and surprisingly, group 2 phenotypes.

#### 3.2.3 From Boolean networks to biological regulatory networks

**Figure 6:**
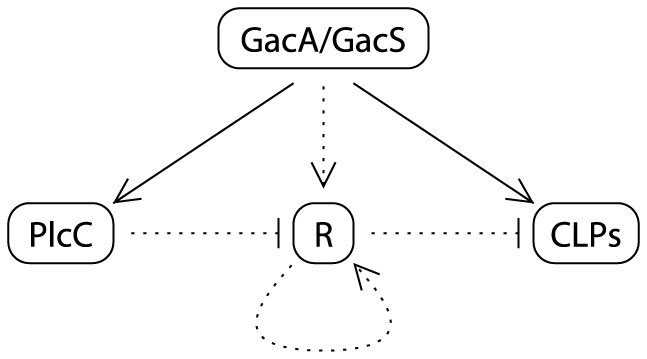
Hypothetic biological regulatory network of CLP production (with negative interactions) deduced from the Boolean model. Circles represent the proteins in the network and edges represent regulatory interaction (bold lines: known interactions, dotted lines: hypothetical interactions). Arrow headed edges represent positive regulatory interaction, and T-headed edges represent negative interaction. PlcC: phospholipase C; CLPs: cyclolipopeptides production; R: unknown regulator (with positive feedback).

The simplest biological regulatory network (with a negative interaction) deduced from the model that accounts for all observations is shown in Figure 6. This regulatory network is likely to be an accurate model, not only for Boolean logic but also for generalized logic [16] where different thresholds or different delays are to be considered. For instance, literature underlines the fact that GacA/GacS system acts on a post-transcriptional level via the Gac/Rsm pathway [20] and via transcriptional mechanisms [5], [21]. If the GacA-PlcC interactions were faster than the GacA-R interactions, the complementation of a 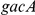 mutant would result in a wild type phenotype. If there are different thresholds of GacA for these two interactions, GacA = 0 when the 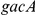 mutation is stable and the system reaches the group 1 attractor. But if there is only a transient decrease of GacA (stochastic event or fast mutation/reversion) with a threshold of GacA sufficient for GacA-R interaction and not for GacA-PlcC interaction, the network would reach the group 2 attractor. So far, this regulatory model explains all our previous observations but the discovery of 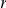 remains the bottleneck of the experimental evidence.

From a biological standpoint, this approach suggests at least three experiments to validate the model with a negative 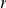 interaction.

According to the dynamics of 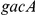 interactions, a 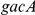 mutant complementation can lead to a wild type phenotype or to a group 2 phenotype. We introduced 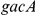 gene in a 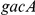 mutant and screened 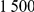 clones for CLP production. All the clones were restored in the wild type phenotype. This could indicate that, in our conditions, GacA-PlcC interactions is favoured instead of the GacA-R interaction.

Secondly, a pulse of R in the wild type strain could lead to group 2 variants. In literature, no negative regulator for CLP production has yet been described. Indeed, identification of such regulators is difficult with the routinely used random mutagenesis. The CLP phenotype of an 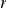 mutant in wild type MFN1032 must be of a wild type according to our model. Only R over-expression in MFN1032 should be detectable in terms of CLP phenotype. We analyzed data from transcriptomic consequences of a 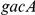 mutation in *Pseudomonas fluorescens* strain Pf-5, a CLP producer [22]. We looked for a putative negative regulator under positive GacA transcriptional control. Among the 5 transcriptional factors positively regulated by GacA during the stationary phase, the only negative identifiable transcriptional regulator belonged to the GntR family. It seems especially relevant, since a 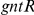 gene was identified downstream 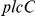 gene in MFN1032. GntR regulators have been reported to maintain their own expression [23], so a positive feedback loop regulating GntR levels may exists. We have promoted overexpression of GntR in the wild type strain MFN1032 by cloning 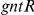 in an expression plasmid with 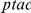 promoter. However this does not impair CLP production, which shows that GntR is not R.

And last, without other valid data from literature, random mutagenesis on 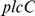 mutant or a group 2 variant may, by a mutation in 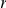, restore wild type CLP production. Such an experiment might help to identify R.

### Conclusion

From a biological point of view, the Boolean approach has proven particularly well suited for this study for several reasons: (i) the qualitative aspect of the observations, which are expressed in terms of the presence/absence of CLPs and PlcC; (ii) the lack of information about the genetic network controlling CLP production; (iii) reproducible but not easily nor understood straightforward observations.

The particularity of our modelling study consists in translating the observations in a formal way, and in computing all the Boolean networks which satisfy the property resulting from this translation, without any assumption on the topology of the considered network (except taking into account the minimal number of genes required). By considering only the minimal Boolean networks (those in which the number of interactions is also minimal) consistent with the observations, we are able to propose new hypotheses concerning the regulatory network of CLP production. There is a strong probability that a yet unknown regulator, endowed with positive self-regulation is negatively controlled by PlcC and negatively controls the production of CLPs.

By contrast, the hypothesis that type 2 variants are due to a mutation/reversion in one of the 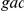 genes (which are known to be highly mutable) is strongly suggested by the models. Finally, the models show the consistency of our hypothesis of a bistability (or epigenetic switch) in CLP production. It is shown more and more often that bistability is a means for bacteria to maintain diversity in their populations. It could account for the diversity of CLP phenotypes observed in *Pseudomonas* strains, in which numerous non-CLP producer strains and CLP producers are found in the same environment [24], [25].

## Supporting Information

Figure S1
**The 12 Boolean networks** 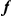 **with three components (** 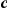 **,** 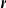 **and** 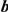 **) that are consistent with property** 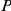 **and with an interaction graph in which** 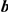 **has no outgoing interaction.** The graphical conventions used to described the interaction graph of each network are as follows: arrows of form 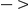 correspond to positive interactions; arrow of form 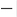 correspond to negative interactions; and arrows of form 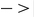 indicate the presence of both positive and negative interaction. Asynchronous state graphs are represented in a 3D-grid, with 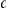 in the vertical axis (first component), 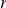 in the horizontal axis (second component), and 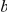 in the third axis (third component).(EPS)
